# National policies for the promotion of physical activity and healthy nutrition in the workplace context: a behaviour change wheel guided content analysis of policy papers in Finland

**DOI:** 10.1186/s12889-017-4574-3

**Published:** 2017-08-02

**Authors:** Tuija Seppälä, Nelli Hankonen, Eveliina Korkiakangas, Johanna Ruusuvuori, Jaana Laitinen

**Affiliations:** 1 Faculty of Social Sciences, University of Tampere, Linna, -33014 Tampere, FI Finland; 2 Finnish Institute of Occupational Health, -00251 Helsinki, FI Finland

**Keywords:** Policy papers, Health promotion, Workplace, Nutrition, Physical activity, Sedentary behaviour, Mechanisms of action, Behaviour change techniques

## Abstract

**Background:**

Health policy papers disseminate recommendations and guidelines for the development and implementation of health promotion interventions. Such documents have rarely been investigated with regard to their assumed mechanisms of action for changing behaviour. The Theoretical Domains Framework (TDF) and Behaviour Change Techniques (BCT) Taxonomy have been used to code behaviour change intervention descriptions, but to our knowledge such “retrofitting” of policy papers has not previously been reported. This study aims first to identify targets, mediators, and change strategies for physical activity (PA) and nutrition behaviour change in Finnish policy papers on workplace health promotion, and second to assess the suitability of the Behaviour Change Wheel (BCW) approach for this purpose.

**Method:**

We searched all national-level health policy papers effectual in Finland in August 2016 focusing on the promotion of PA and/or healthy nutrition in the workplace context (*n* = 6). Policy recommendations targeting employees’ nutrition and PA including sedentary behaviour (SB) were coded using BCW, TDF, and BCT Taxonomy.

**Results:**

A total of 125 recommendations were coded in the six policy papers, and in two additional documents referenced by them. *Psychological capability, physical opportunity,* and *social opportunity* were frequently identified (22%, 31%, and 24%, respectively), whereas *physical capability* was almost completely absent (1%). Three TDF domains (*knowledge, skills*, and *social influence*) were observed in all papers. Multiple intervention functions and BCTs were identified in all papers but several recommendations were too vague to be coded reliably. Influencing individuals (46%) and changing the physical environment (44%) were recommended more frequently than influencing the social environment (10%).

**Conclusions:**

The BCW approach appeared to be useful for analysing the content of health policy papers. Paying more attention to underlying assumptions regarding behavioural change processes may help to identify neglected aspects in current policy, and to develop interventions based on recommendations, thus helping to increase the impact of policy papers.

**Electronic supplementary material:**

The online version of this article (doi:10.1186/s12889-017-4574-3) contains supplementary material, which is available to authorized users.

## Background

There is some evidence to suggest that health behaviour interventions that are designed on the bases of behavioural theories are more effective than those that are not [1, 2]. Such interventions allow more systematic and evidence-based ways of identifying relevant behavioural determinants and appropriate intervention strategies [3], as well as more effective evaluation because the proposed mechanisms by which they operate are made explicit. The same may be true for health policy. Therefore, governments have displayed an increasing interest in utilizing behavioural scientific evidence when designing public policy [4]. Health policy is defined by the World Health Organization as “decisions, plans, and actions that are undertaken to achieve specific health care goals within a society” [5]. Policies are a means for generating and/or supporting the implementation of health behaviour change interventions [6], which are a set of activities designed to bring about change [3]; thus policies are crucial for the interventions’ implementation and outcomes [3, 7–9]. Policy papers are a channel to disseminate recommendations and guidelines for health behaviour change, such as national nutrition recommendations. Mechanisms of change described or implied in policy papers have not been studied to our knowledge. Such an analysis would be essential to identify gaps with regard to behavioural science evidence in policy recommendations.

The Behaviour Change Wheel (BCW) [10] and Theoretical Domains Framework (TDF) [11, 12] are potential tools for such analyses. These frameworks have been utilized to retrospectively analyse the elements of planned and implemented interventions [13, 14], but to our knowledge such “retrofitting” to policy papers has not been comprehensively reported. If suitable for policy paper analysis, these frameworks may aid in identifying important content of policy papers with regard to behaviour change, and may offer tools to further develop policymaking, and implement policy into practice.

BCW is a theory- and evidence-based model for characterizing and designing behaviour change interventions [16] (see Michie et al. [10, 15] for detailed description of the model). BCW aims to be a comprehensive and coherent framework that links interventions to an overarching model of behaviour, COM-B (capability (C), opportunity (O), motivation (M), and behaviour (B)). COM-B postulates that three factors are necessary and sufficient prerequisites for the performance of a specified volitional behaviour: *psychological* and *physical capability* (i.e. the individual’s psychological and physical capacity to engage in the activity concerned, including the necessary knowledge and skills), *social* and *physical opportunity* (i.e. all the factors that lie outside the individual that make the behaviour possible or prompt it), and *reflective* and *automatic motivation* (i.e. all the brain processes that energize and direct behaviour, including goals, emotional responses, analytical decision-making, and habitual processes). Furthermore, *opportunity* and *capability* can influence *motivation,* and behaviour might influence all the other components. [10, 15] To be effective, behaviour change interventions should target those components that are most likely to influence the target behaviour of the target population in a specified context [3]. For example, a workplace intervention to reduce sedentary behaviour should target capability and opportunity if the target employees are motivated to add physical activity during the workday but the work environment does not support their behaviour change and they do not know how to use the new standing desks. BCW recognizes nine non-overlapping categories of activity aimed at changing behaviour, called intervention functions (IF) (education, persuasion, incentivization, coercion, training, restriction, environmental restructuring, modelling, and enablement), and seven non-overlapping policy categories (PC) (communication/marketing, guidelines, fiscal policy, regulation, legislation, environmental/social planning, and service provision) that enable or support these IFs [10, 15]. IFs may be conducted by using various behaviour change techniques (BCT) [15, 16]. For example, educational intervention may include instructions on how to perform the behaviour, demonstration of the behaviour, and behaviour rehearsal.

The BCW approach can be used to both develop and “retrofit” interventions [10]. COM-B helps designers and evaluators of interventions to assess where the intervention is or should be targeted. The differentiation of IFs helps to select or evaluate possible means of behaviour change. TDF extends the COM-B analysis and helps to evaluate the theoretical bases of planned and conducted interventions [11]. TDF aims to capture the optimal structure, content, and labels of related theoretical constructs, and the most recent version of TDF includes 14 domains [10]. Theoretical domains can be linked to a specific component of COM-B [10]. For example, theoretical constructs related to *social influences* (one of the 14 theoretical domains), such as *social support* and *group identity,* are relevant for influencing the *social opportunity* component of behaviour.

Workplace health promotion (WHP) is a topical context in which to investigate possible gaps with regard to behavioural science evidence and policy recommendations and to analyse the suitability of BCW and TDF for policy paper analysis. In Finland, researchers and policymakers alike perceive workplaces as prominent sites for health promotion [17–19]. Negative health developments, such as increasing obesity and decreasing physical condition, in tandem with pressure to extend work careers, are perceived to require investment in changes in employees’ health behaviour [20]. Physical activity (PA) is perceived as especially important in the strategy papers. Reducing excessive sedentary behaviour (SB) has recently also been raised as one of the key issues in WHP [21], and Finland is one of the first countries to launch a national strategy for the reduction of SB. Additionally, catering services are focus of national policy and WHP, as they have an essential role in healthy nutrition during working hours [22].

Research evidence suggests that healthy behaviours are related to better work ability and safety at work [23–25]. Several strategy papers therefore highlight the importance of close collaboration between occupational healthcare (OHC) and workplaces for the promotion of work ability and the reduction of disability at work through health behaviour changes [19, 26]. In Finland, employers are responsible for employees’ health and safety at work, and they are obliged to arrange OHC for employees. WHP is conducted in collaboration among employers, employees, health and safety organizations, and OHC [27]. Thus it can be suggested that policy papers should offer guidelines and recommendations at the organizational level to promote health. It seems, however, that the implementation of policy papers may not have succeeded optimally, as reviews of WHP interventions have found at best moderate positive effects on nutrition, PA, and SB, and the conclusions are limited by the low quality and number of reported interventions [21, 28–36].

In this study we investigate the kinds of mechanism of action described or implied in Finnish health policy papers that aim to promote healthy nutrition and PA, and reduction of SB in the worksite. More precisely, we explore which mechanisms of action or intervention strategies, as characterized by COM-B, TDF, IFs, and BCT Taxonomy, are emphasized or lacking in policies and whether these differ between healthy nutrition and PA. We also evaluate, the extent to which the policy recommendations target the individual level, the community level, or the environment. As a secondary purpose, we assess whether the BCW approach, including COM-B, TDF, IFs, and BCT Taxonomy, are suitable for analysing policy papers.

## Methods

### Materials and data selection

We systematically searched the websites of national ministries and research institutes working under ministries for all national-level health policy papers regarding the promotion of healthy nutrition and PA in September 2016. The inclusion criteria were that the policy papers should: 1) be at the national level (i.e. not local or international); 2) be related to health (e.g. not environmental policy); 3) be the most recent version of the policy paper, or related to an ongoing policy programme at the time of the search; 4) include nutrition and/or physical activity; 5) include, but not necessarily be restricted to, a workplace context. Using these criteria, six policy papers were found. We also analysed the content of all the other papers (Ref [1] and Ref [2] in Table 1) that were referred to in the policy recommendations of the selected papers, and that focused on healthy nutrition or PA in the worksite. Finally, we consulted clinical practice guidelines and documents (Tec 1, Tec 2 and Tec 3 in Table 1) that described the content of the recommended tools (e.g. motivating interview; physical activity prescription). The papers and documents are summarized in Table 1. Each paper and data unit selection (i.e. recommendation) is described in Additional file 1. Data unit selection was independently conducted by two coders (TS and EK). After this independent selection, the coders compared data sets and made sure that all recommended means to change the target health behaviours were included and that every recommendation was only included once per paper in the data set.

**Table 1:** Selected primary and secondary policy papers and sources of technical terms

Policy paper	Publisher	Year	Pages	Target
1. Principles of good occupational healthcare practice guide	Ministry of Social Affairs and Health/Finnish Institute of Occupational Health	2014	317	OHC professionals
2. National nutrition recommendations	National Nutrition Council	2014	60	Healthcare, catering and food industry professionals, authorities, and public health organizations
3. Guidelines of the working group to monitor and develop mass catering services	Ministry of Social Affairs and Health	2010	82	Administration, private sector, social partners, NGOs
4. National strategy for physical activity promoting health and well-being 2020	Ministry of Social Affairs and Health	2013	62	National and local actors
5. Action plan of the National Obesity Programme 2012–2015	National Institute for Health and Well-being	2013	59	State and municipal administration, healthcare, early childhood education, education, sports department, technical and community planning, educational organizations, NGOs, defence forces, employers and trade unions, food industry, trade, restaurants and catering, media, research institutions
6. National strategy for the reduction of sedentary behaviour	Ministry of Social Affairs and Health	2015	44	Children, adolescents, parents, instructors, teachers, day care, schools, municipalities, students, employees, study communities, work communities, aged, care services
Ref [1]. National action plan for walking and cycling 2020	Finnish Transport Agency	2012	76	State, municipal and private sector actors
Ref [2]. Recommendations for physical activity promotion in municipalities	Ministry of Social Affairs and Health	2010	24	Municipality management, ports department, social and health department, youth department, culture and library department, technical departments
6. Tec 1. Clinical practice guidelines for physical activity	Finnish Medical Society Duodecim	2016	27	Healthcare professionals
7. Tec 2. Clinical practice guideline for obesity care	Finnish Medical Society Duodecim	2013	27	Healthcare professionals
Tec 3. Physical activity prescription	UKK Institute	2013	23	Healthcare professionals

Policy papers 1–6 are the primary papers investigated. Paper Ref [1] was referred to in paper 4, and paper Ref [2] was referred to in papers 4 and 5. The content of technical terms presented in policy papers was checked in the documents Tec 1, Tec 2, and Tec 3

### Coding

We built a coding matrix based on COM-B, TDF, and BCTs. The coding instructions are presented in Additional file 2. The first code identified the target health behaviour: nutrition, or PA including SB. The second code identified the target: individual (i.e. individual behaviour), environment (i.e. physical environment), or community (i.e. social environment) (examples are presented in Table 4). This study only includes policy recommendations targeted directly or indirectly at employees through the physical or social environment (examples are presented in Tables 2 and 3); recommendations solely targeting providers or the system, without any obvious link to individual behaviour change, are excluded (e.g., “Multi-professional collaboration is enhanced at regional level”). In this way we could focus on the relevant target population in respect of our target behaviour (i.e. nutrition, PA, and SB of employees). Next, the codes for the BCW approach were identified: COM-B components (code 3), TDF (code 4), IFs (code 5), and BCTs (code 6). The recommendations that were too general or vague to be reliably coded were coded “too vague to be coded”.

**Table 2:** Identified mechanisms of action recommended in health policy papers

Mechanisms of action				
COM-B	N (%)	TDF	N (%)	*Example*
Psychological capability	34 (22)	Knowledge	27 (14)	*Employees are given written instructions, handouts, and material for health promotion.*
		Skills	12 (6)	*A customer needs to be guided in putting together a recommended meal.*
		MAD	7 (4)	*Healthy choices are positioned so that they are easy to select from the service line.*
		BR	6 (3)	*Recognize your habits by measuring your activity and steps*.
Physical capability	2 (1)	Skills	2 (1)	*The OHC professional makes sure that an employee has the knowledge and skills needed for health and safety at work and for the promotion of work ability*.
Physical opportunity	47 (31)	Env.	48 (25)	*Local actors enhance opportunities for varying working postures by activating furniture and equipment.*
Social opportunity	37 (24)	Social Influences	38 (20)	*Influencing personal health-related attitudes, lifestyles, and risk behaviour by discussion.*
Reflective motivation	24 (16)	S/P id	1 (1)	*Seminar organizer may encourage the audience into a standing ovation - you can be the Trendsetter too!*
		B Cap	6 (3)	*Monitoring and evaluation of employee lifestyle choices.*
		Optimism	1 (1)	*Positive aspects and even small advances are always identified first.*
		B Con	17 (9)	*Physically active lifestyle and healthy nutrition are highlighted as a part of health and well-being.*
		Intentions	8 (4)	*Needs for change are identified as part of lifestyle counselling.*
		Goals	4 (2)	*Define the goals of physical activity*.
Automatic motivation	10 (6)	S/P id	1 (1)	*Public sector employees also need to lead by example in the promotion of walking and cycling.*
		Optimism	0 (0)	-
		Reinforcement	9 (5)	*Food must also be tasty and served in an appealing manner.*
		Emotion	2 (1)	*The physically inactive are enabled to experience physical activity as a source of pleasure.*
Total	154 (100%)	Total	189 (100%)	

Numbers are frequencies. Numbers in parentheses are percentages. TDF domain abbreviations: *MAD* memory, attention, and decision processes; *BR* behavioural regulation; *Env.* environmental context and resources; *S/P id* social/professional role and identity; *B Cap* beliefs about capability; *B Con* beliefs about consequences

**Table 3:** Identified IFs and BCTs recommended in policy papers

Intervention function N (%)									
Education 27 (23)	Persuasion 15 (13)	Incentivization 6 (5)	Coercion 2 (2)	Training 2 (2)	Restriction 5 (4)	Env. restructuring 34 (29)	Modelling 1 (1)	Enablement 24 (20)	Total 116 (100%)
BCTs	Examples							N (%)	
Goal-setting (behaviour)	*Goals and actions are recorded in personal health plans in order of importance.*							3 (2)	
Goal-setting (outcome)	*Goals and actions are recorded in personal health plans in order of importance.*							1 (1)	
Action-planning	*Employees are given a copy of the personal health plan.*							2 (1)	
Discrepancy between current behaviour and goal	*Pointing out the discrepancy between current behaviour and important goals for the patient is aimed at.*							3 (2)	
Behavioural contract	*Goals and actions are recorded in personal health plans in order of importance.*							1 (1)	
Monitoring of behaviour^a^	*Achievement of goals needs to be monitored.*							3 (2)	
Feedback on behaviour	*Monitoring and evaluation of employee lifestyle choices.*							3 (2)	
Self-monitoring^a^	*Customers are supported in self-monitoring*.							1 (1)	
Self-monitoring of behaviour	*For example, a food diary can be used for planning, following, and monitoring a diet.*							4 (2)	
Social support (unspecified)	*Encouragement to make health promoting choices.*							18 (11)	
Social support (emotional)	*The patient is not criticized but understanding for their reactions is shown and emotions are accepted.*							4 (2)	
Instructions on how to perform the behaviour	*Information can be given by presenting model meals that are compiled of day supply.*							20 (12)	
Information about antecedents	*Factors and resources supporting the change are identified*.							1 (1)	
Information about health consequences	*Physically active lifestyle and healthy nutrition are highlighted as a part of health and well-being.*							11 (7)	
Demonstration of the behaviour	*Supervisors, lead by example*.							2 (1)	
Prompts/cues	*The heart symbol helps to select suitable foods for the recommended diet.*							3 (2)	
Behavioural practice/rehearsal	*For example, selecting healthy foods can be rehearsed in practice.*							7 (4)	
Behaviour substitution	*Instead of sitting, various activities can be done while standing or lightly moving. Lunch, coffee breaks and screen work also work well while standing.*							2 (1)	
Habit formation	*Continue to conduct the change persistently, because new habits turn into practice within couple of weeks or months.*							5 (3)	
Habit reversal	*Change your daily routines one by one so that you reduce sedentary behaviour and take breaks during long sedentary periods.*							1 (1)	
Generalization of target behaviour	*Change your daily routines one by one so that you reduce sedentary behaviour and take breaks during long sedentary periods.*							1 (1)	
Material incentive	*A tax-free compensation for commuting by bicycling paid by the employer.*							4 (2)	
Social reward	*Monitoring and evaluation of employee’s lifestyle choices.*							1 (1)	
Reduce negative emotions	*Change your routines one by one. Keep changing persistently. Be moderate in everything. Heavy work might warrant a rest by sitting or even lying down.*							3 (2)	
Restructuring the physical environment	*Healthiness of catering for meetings is important to take into account.*							38 (24)	
Restructuring the social environment	*Write down rules for reduction and breaking of sedentary behaviour together with different stakeholders in the work community.*							11 (7)	
Avoidance of exposure to cues for the behaviour	*Avoidance of exposure to unnecessary temptations (*e.g.*energyintensive snack foods are not purchased for the home).*							1 (1)	
Adding objects to the environment	*Local actors enhance opportunities for varying work postures by activating furniture and equipment.*							4 (2)	
Identification of self as role model	*Seminar organizer may encourage the audience into standing ovation - you can be the Trendsetter too!*							2 (1)	
Incompatible beliefs	*Pointing out the discrepancy between current behaviour and important values for the patient is aimed at.*							1 (1)	
Verbal persuasion about capability	*Identification and reinforcement of personal strengths are aimed at. Positive aspects and even small advances are always identified first.*							2 (1)	
Total number of observed BCTs								163 (100%)	

Numbers are frequencies. Numbers in parentheses are percentages. ^a^More general formulation than in BCT Taxonomy (v1)

Policy papers were coded one by one, and consistency of coding was secured through comparison. The iterative coding process aimed for consensus among the coders through the comparison of independent coding (TS, EK, NH), discussion, and the consultation of available materials such as definitions, examples, previous studies, and coding principles. Unclear cases were further discussed with researchers familiar with the frameworks in use and coding.

## Results

The results and examples from the data in respect of COM-B and TDF are presented in Table 2. More than half (55%) of the recommendations were categorized as *opportunity* (31% physical and 24% social opportunity). *Psychological capability* was also common (22%), but mentions of *physical capability* were almost completely absent (1%). One fifth of the observations were coded as r*eflective* (16%) and *automatic* (6%) *motivation*. In line with this, *environmental context and resources* (25%), *social influences* (20%), and *knowledge* (14%) formed the majority of the theoretical domains identified. None of the domains was absent, but *social role and identity* (*n* = 2), *goals* (*n* = 4), *optimism* (*n* = 1), and *emotion* (*n* = 2) were relatively rare.

Small differences in COM-B aspects and TDF domains were observed in relation to the type of behaviour: the most frequent code for nutrition was *psychological capability* (28%), which was less common for PA (18%). The most common COM-B mechanism for PA was *physical opportunity* (27%), which was almost as frequently coded for nutrition (24%). The difference was mainly constituted by two domains: *knowledge* (17%) and *memory, attention and decision processes* (7%) were coded slightly more frequently among recommendations for nutrition than for PA (13% and 0% respectively). Results in terms of nutrition and PA are presented in Additional file 3: Table S1.

Results concerning IFs and BCTs are presented in Table 3. Seventeen per cent of the recommendations were general and included only what but not how (e.g. “employees are activated to self-monitor...”); in these cases no IF was coded. All papers recommended a range of interventions. The most frequently coded IFs were *environmental restructuring* (29%), *education* (23%), and *enablement* (21%). All nine IFs were identified but, *modelling* (*n* = 1), *training* (*n* = 2), *coercion* (*n* = 2), and *restriction* (*n* = 5) were relatively rare. Educational interventions were identified somewhat more frequently for nutrition (31%) than for PA (23%). In total, 31 different BCTs were identified. Additional material for professionals (see Table 1) was consulted during the coding of technical terms, even though the recommendations never cited this material. Three BCTs were identified in all papers: *instructions on how to perform the behaviour*, *social support (unspecified)*, and *information about health consequences* (see Additional file 4: Table S2). The majority of identified BCTs belonged to the BCT clusters *antecedents* (*n* = 55), *social support* (*n* = 22), and *shaping knowledge* (*n* = 21). No BCTs were identified from the BCT clusters *scheduled consequences* or *covert learning*.

In terms of socio-ecological levels, the primary targets of recommendations are presented in Table 4. The majority of recommended actions for employee health behaviour change targeted individual behaviour (46%) and suggested changes to the environment (44%); only a minority suggested changes to the community (e.g. culture) (10%). The relative frequency of the environment was somewhat higher in relation to nutrition (47%) than to PA (30%). Community-targeted recommendations were rare in nutrition-related (*n* = 2) but slightly more frequent in PA-related (14%) recommendations. Results from each policy paper are presented in Additional file 5: Table S3.

**Table 4:** Identified targets in policy recommendations

Target	Examples	Total(%)	Nutr.(%)	PA(%)
Individual	*Use stairs whenever possible.* *Monitoring and evaluation of employee’s lifestyle choices.*	57 (46)	39 (50)	46 (55)
Community	*Rethink meeting practices to reduce sedentary behaviour at your workplace.*	13 (10)	2 (3)	12 (14)
Environment	*Healthiness of catering for meetings is important to take into account.*	55 (44)	37 (47)	25 (30)
Total		125 (100%)	78 (100%)	83 (100%)

Numbers are frequencies. Numbers in parentheses are percentages. *Nutr.* nutrition, *PA* physical activity. The total is smaller than the sum of Nutr. and PA because parts of the recommendations (i.e. focusing on outcomes or health behaviour in general) were double coded

## Discussion

The investigated policy papers emphasized *opportunity* and *psychological capability* in the promotion of PA and healthy nutrition, whereas recommendations focusing on *physical capability* were almost absent. PA skills are an essential part of basic education in Finland, and on average citizens can be estimated to have sufficient skills to engage in health-promoting PA. On the other hand, the skills needed to prepare healthy meals have a minor role in basic education. The findings also suggest that recommendations are based on or aligned with a limited set of theories of behaviour change. Although *emotions* [37, 38], *optimism* [39, 40], and *identity* [41] have all been found to be associated with health behaviours, they were only identified in one or two papers.

All papers recommended multiple IFs, but only *education*, *enablement*, and *persuasion* were identified in all eight papers and *environmental restructuring* in all but one. WHP studies have shown support for multicomponent interventions, even though most have been educational and environmental interventions [21, 28–30, 33–35, 42]. Furthermore, recommendations targeting the community were uncommon, and nearly absent for nutrition. This is a clear limitation, as evidence suggests that social environment is important in WHP [43].

In total 31 BCTs were identified. The most frequently identified BCTs were *information about health consequences*, *instructions on how to perform the behaviour*, *restructuring the physical environment*, and *social support*. The findings imply that policy papers rely heavily on the assumption that providing information about health consequences and environmental changes are the most effective tools. However, there is evidence to show that health behaviours are motivated by a broader range of perceived benefits than health alone [44]. In light of behavioural science evidence, some clear misunderstandings were also identified. For example, an underlying assumption in some recommendations was that providing information to employees was a sufficient strategy to promote behaviour change. Further, *social support* was almost entirely limited to encouragement and counselling from healthcare professionals, and mentions of practical support were also absent despite evidence suggesting that other sources of social support are also beneficial for WHP [21, 36]. Finally, for PA and nutrition behaviours, control-theory related BCTs (e.g. goal setting, self-monitoring, reviewing goals) have been identified as effective [45], but these received only minor emphasis in the policy papers investigated.

As this was among the first studies using BCW and the retrofit approach to policy papers, there were some newly encountered challenges in the analysis. First, in most cases, COM-B components and TDF domains were inseparably linked to the recommended IF. For example, recommended changes in *physical environment* were mapped onto *physical opportunity* and *environmental context and resources*. However, in some cases IFs were too vague to be coded but the TDF component was codable (e.g. “The physically inactive are enabled to experience physical activity as a source of pleasure”). Second, in some cases; the TDF domains *environmental context and resources* and *social influences* appeared to overlap. For example, we perceived that changes in organizational practices included constructs from both domains, and we coded accordingly. Moreover, we categorized *modelling* of non-human models (i.e. a plate model) to the less optimal domain *knowledge* because the construct *modelling* is defined as an interpersonal process in TDF. Third, the BCW approach is greatly applicable to the assessment of individual-level interventions, but its current version has limitations in describing mid-level interventions conducted in organizations. For example, these interventions might include changes in organizational strategy, culture, or leadership, which are all substantial elements of WHP [46]. In this study, the IF code for this level, *environmental restructuring,* might not sufficiently differentiate between the specific characteristics of such activities. Fourth, the coding of BCTs was very challenging, mainly due to the unspecific content of the policy papers. Furthermore, we coded diverse actions as the BCT *restructuring the physical environment*, such as changes in services, pricing (when coding the “material incentive” or “reward” was not appropriate), placement, and availability. This BCT could be further divided to better reflect the actual content of environmental restructuring.

This study has some limitations. First, we only investigated recommendations targeting employee behaviour change, and excluded recommendations focusing on providers and systems that had no obvious link to employee behaviour change. Second, the papers included references to further complex interventions, such as counselling by a nutrition therapist, which were likely to include a large number of BCTs; but since these were not specified in the policy documents, they were not coded. Thus our results may represent conservative estimates of the presence of IFs or BCTs. Finally, the “dose” or volume of the suggested policies is not directly derivable from our calculations. However, the policy papers themselves could be quite vague on the suggested relative volumes.

This study has some practical implications for developing policy papers. First, the design of effective national policy involving any behaviour change may benefit from a comprehensive behavioural analysis [10] of both the target population and the policy providers and implementers, and from a consultation of scientific evidence to identify effective interventions. This information, including the assumed mechanisms of action at play, could be explicitly conveyed in the policy paper, which would aid both evaluation and implementation. This might include balancing and coordinating of activities that should ideally be implemented in consort with each other for the desired impact. Second, recommendations should be clear in terms of who is supposed to deliver what to whom and how. Almost a fifth (17%) of the recommendations failed to explicate the means for achieving the defined goals. Such lack of specificity provides little practical guidance for WHP, and might impair the quality of interventions based on the recommendations [3]. On the other hand, it may be argued that the function of policy papers is not to stipulate particular active ingredients of interventions, as it might be difficult to specify intervention contents for all the different contexts in national-level policy papers. However, in line with recently acknowledged calls to employ behavioural science more efficiently in public policy [4], this may be exactly what policy papers should move towards: better specifying courses of action that have a strong evidence base, leaving less room for interpretation among the intervention’s developers.

Suggestions for future research include similar analyses of WHP policy papers in other countries, as well as of other policies. Policy papers may currently underuse behaviour change science, relying mostly on information provision. Furthermore, it would be important to study upper- or system-level recommendations and map those onto the behaviour change interventions suggested in policy papers. It has been argued, for example, that conservative politicians may emphasize strategies that place responsibility at the level of individuals and rational choices, and that liberal or left-wing politicians may attempt to influence policies through stronger government regulation [47]. Thus in future such an analysis would enable the empirical investigation of longitudinal changes in the assumed underpinnings of behaviour and the preferred change strategies in policies, both within particular countries and in cross-national comparisons.

## Conclusions

Our analysis shows that frameworks initially developed for the planning and assessment of behaviour change interventions and implementation can be used to assess the content of policy papers on health promotion. Retrofitting revealed the strengths, limitations, and development needs of policy papers and suggested initiatives for further development of the BCW approach.

## Additional files

Additional file 1: Description of selected policy papers and data unit selection strategy. (DOCX 20 kb)

Additional file 2: Coding instructions. (DOCX 19 kb)

Additional file 3: Table S1. Identified mechanisms of action for nutrition and PA recommended in health policy papers. (DOCX 17 kb)

Additional file 4: Table S2. Identified intervention content recommended in policy papers for nutrition and PA. (DOCX 22 kb)

Additional file 5: Table S3. Frequencies of different targets in recommendations for each policy paper. (DOCX 14 kb)

## Abbreviations

BCT: Behaviour change technique
BCW: Behaviour Change Wheel
COM-B: Capability-opportunity-motivation-behaviour
IF: Intervention function
OHC: Occupational healthcare
PA: Physical activity
PC: Policy category
SB: Sedentary behaviour
TDF: Theoretical Domains Framework
WHP: Workplace health promotion
