# Supplementary material for: National policies for the promotion of physical activity and healthy nutrition in the workplace context: a behaviour change wheel guided content analysis of policy papers in Finland
Source: BMC Public Health. 2017 Aug 2;18:87. doi: 10.1186/s12889-017-4574-3 (PMC5540493; doi:10.1186/s12889-017-4574-3)
Supplement: Supplementary file 1 — Description of selected policy papers and data unit selection strategy. (DOCX 20 kb) [file 12889_2017_4574_MOESM1_ESM.docx]

Additional file 1

Description of selected policy papers and data unit selection strategy

1. Principles of good occupational health care practice guide: describes how to apply in practice the Government Decree on Principles of Good Occupational Healthcare Practice, the Content of Occupational Healthcare and the Qualifications of Professionals and Experts (No. 708/2013). The decree is laid down under the Occupational Healthcare Act (No. 1383/2001) and is the foundation for OHC practice including health behaviour promotion. We selected all recommended actions for the promotion of health behaviour, excluding parts that explicitly focused on health behaviours other than PA, SB, or healthy nutrition.
2. National nutrition recommendations: the foundation of national food and nutrition policies. The paper includes evidence-based recommendations for foods and nutrients and suggestions for various providers on implementation. We selected all recommended actions for the promotion of healthy nutrition whenever they mentioned workplaces, workplace representatives, OCH, healthcare, and catering services as targets or providers.
3. Guideline of the working group to monitor and develop mass catering services: implements national nutrition recommendations in the context of mass catering services. The guidelines briefly present five broad recommendations for actions to promote the availability, attractiveness, and use of healthy catering services, a suggestion for the use of indicators, and a suggestion for systematic monitoring, which are further explicated throughout the paper. We selected all recommended actions for the promotion of healthy nutrition that mentioned workplaces, workplace representatives, OHC, healthcare, and catering services as targets or providers.
4. National strategy for physical activity promoting health and well-being 2020: presents goals and actions for different stakeholders to promote citizens’ PA. The programme aims to reduce SB, increase PA, highlight the preventive role of PA, and strengthen the role of PA in society. The paper presents four broad goals and lists means separately for national, regional, and local actors. We selected all recommended actions for the promotion of PA and reduction of SB that mentioned workplaces, workplace representatives, OCH, healthcare, and catering services as targets or providers.
5. Action plan of the National Obesity Programme 2012-2015 presents goals and means for various providers to reduce obesity and promote healthy nutrition and PA. Suggested means for different providers are listed at the end of the paper. We selected all recommended actions for the promotion of PA and healthy nutrition targeted at workplaces, workplace representatives, OHC, healthcare, and catering services.
6. National strategy for the reduction of sedentary behaviour: presents means for different population groups and providers to reduce SB and increase PA. Suggested means for different target groups and providers are presented under separate headlines. We selected all recommended actions for the promotion of PA and reduction of SB targeted at workplaces, workplace representatives, OHC, and healthcare. Suggestions for catering services are not presented. Unlike in other papers, in this paper employees constitute one target group (i.e. recommendations are presented directly to employees), which means that the paper can be considered an employee-targeting intervention. We therefore analysed all the other elements in the paper that could be considered part of the intervention (e.g. information about health consequences) and included these in the data.

Ref 1. National action plan for walking and cycling 2020: not included in the selected policy papers because it was not a health policy paper but was based on climate politics. However, one recommendation in a selected paper (National strategy for physical activity promoting health and well-being 2020) was to take into account the recommendations of this action plan. We therefore selected all the recommendations for the promotion of physical activity targeted at workplaces and workplace representatives presented in the action plan.

Ref 2. Recommendations for physical activity promotion in municipalities: not included in the selected policy papers because it was based on a Local Government Act and targeted at decision makers in local governments. However, a recommendation in two selected papers (National strategy for physical activity promoting health and well-being 2020; Action plan of national obesity programme 2012-2015) was to take into account the recommendations in this paper. We therefore selected all the recommendations for the promotion of physical activity insofar they focused on workplace contexts.

Tec 1. Clinical practice guidelines for physical activity: consulted for the content of technical terms presented in the recommendations of selected papers.

Tec 2. Clinical practice guidelines for obesity care: consulted for the content of technical terms presented in the recommendations of selected papers.

Tec 3. Physical activity prescription: consulted for the content of the tool. The tool was recommended in the National strategy for physical activity promoting health and well-being 2020.
