# Supplementary material for: National policies for the promotion of physical activity and healthy nutrition in the workplace context: a behaviour change wheel guided content analysis of policy papers in Finland
Source: BMC Public Health. 2017 Aug 2;18:87. doi: 10.1186/s12889-017-4574-3 (PMC5540493; doi:10.1186/s12889-017-4574-3)
Supplement: Supplementary file 2 — Coding instructions. (DOCX 19 kb) [file 12889_2017_4574_MOESM2_ESM.docx]

Additional file 2

Coding instructions

General guidelines

- Only code recommendations applied in workplace health promotion contexts.
- Only code recommendations targeting nutrition or physical activity (including sedentary behaviour).
- Only code each intervention in terms of nutrition or physical activity once in each paper.
- Code recommendations from an employee’s perspective.
- Code “Too vague to be coded” if the recommendation is too vague to be coded reliably.

Code 1: Target health behaviour nutrition/physical activity

- Code NUTRITION if nutrition is mentioned in the recommendation.
- Code PHYSICAL ACTIVITY if physical activity or sedentary behaviour is mentioned in the recommendation.
- Code NUTRITION and PHYSICAL ACTIVITY if the target behaviour is lifestyle in general and the context supports that interpretation.
- Code NUTRITION and PHYSICAL ACTIVITY if the target is an outcome (e.g. obesity) and the context supports that interpretation.

Code 2: Target individual/community/environment

- Code INDIVIDUAL if the recommendation is directly presented to an employee (e.g. *Use stairs whenever possible*).
- Code INDIVIDUAL if the recommendation is directly presented to a provider whose action targets an employee (e.g. *Encourage to make healthy choices*).
- Code COMMUNITY if the recommendation targets a work community (e.g. *Rethink meeting practices to reduce sedentary behaviour in your workplace*).
- Code ENVIRONMENT if the recommendation targets a provider or system but clearly implies intervention in individual behaviour as well (e.g. *Professionals’ abilities to identify and support people suffering from eating disorders are enhanced*).
- Code ENVIRONMENT if the recommendation targets physical environment but clearly implies intervention in individual behaviour as well (e.g. *An extensive service menu is offered including web, phone, and group counselling to support health behaviour changes*).

Code 3: COM-B

- Code PSYCHOLOGICAL CAPABILITY if the recommendation aims to influence individual psychological capacity to engage in the target behaviour.
- Code PHYSICAL CAPABILITY if the recommendation aims to influence physical capacity to engage in the target behaviour.
- Code SOCIAL OPPORTUNITY if the recommendation aims to influence social factors outside the individual that enhance opportunity to engage in the target behaviour or prompt it.
- Code PHYSICAL OPPORTUNITY if the recommendation aims to influence physical factors outside the individual that enhance opportunity to engage in the target behaviour or prompt it.
- Code REFLECTIVE MOTIVATION if the recommendation aims to influence reflective brain processes that energize or direct the target behaviour.
- Code AUTOMATIC MOTIVATION if the recommendation aims to influence automatic brain processes that energize or direct the target behaviour.
- Code “Too vague to be coded” if the recommendation is not explicit enough to be coded reliably.
- Make sure that the coded COM-B is mapped onto TDF domains as presented in Cane et al. (2012) Table 3.

Code 4: TDF

- Select the most appropriate domain following the definitions of domains presented by Cane et al. (2012).
- Code “Too vague to be coded” if the recommendation is not explicit enough to be coded reliably.
- Make sure that the coded TDF domain is mapped onto the component of COM-B as presented in Cane et al. (2012) Table 3.

Code 5: Intervention function

- Select the most appropriate intervention function following the definitions of functions presented by Michie et al. (2011).
- Code “Too vague to be coded” if the recommendation is not explicit enough to be coded reliably.
- Make sure that the selected intervention function is linked to the component of the COM-B as presented in Michie et al. (2011) Table 2.

Code 6: BCT

- Only code BCTs that are directly applied to nutrition, physical activity, or sedentary behaviours.
- Only code BCTs that are applied to employees.
- Select the most appropriate BCTs following BCT Taxonomy v1, (Michie et al. 2013).
- Take care when distinguishing between BCTs that differ in terms of behaviour change type.
- Code technical terms and packages of BCTs that map onto BCTs in the Taxonomy. Also use the available official material (e.g., guidelines for clinical practice).
- Code BCT even though the action verb is missing if the recommendation is detailed enough to be coded reliably.
- Leave empty if there is none.
- Code “Too vague to be coded” if the recommendation is not explicit enough to be coded reliably.

References

Cane J, O’Connor D, Michie S. Validation of the theoretical domains framework for use in behavior change and intervention research. Implementation Science 2012;7:37.

Michie S, van Stralen MM, West R: The behavior change wheel: a new method for characterising and designing behavior change interventions. Implementation Science 2011; 6:42.

Michie S, Richardson M, Johnston M, Abraham C, Francis J, Hardeman W, Eccles MP, Cane J, Wood CE. The behavior change technique taxonomy (v1) 93 hierarchically clustered techniques: building an international consensus for the reporting of behavior change interventions. Ann Behav Med 2013; 46(1):86-95.
