# Supplementary material for: National policies for the promotion of physical activity and healthy nutrition in the workplace context: a behaviour change wheel guided content analysis of policy papers in Finland
Source: BMC Public Health. 2017 Aug 2;18:87. doi: 10.1186/s12889-017-4574-3 (PMC5540493; doi:10.1186/s12889-017-4574-3)
Supplement: Supplementary file 3 — Identified mechanisms of action for nutrition and PA recommended in health policy papers. (DOCX 17 kb) [file 12889_2017_4574_MOESM3_ESM.docx]

Additional file 3

Table S1

Identified mechanisms of action for nutrition and PA recommended in health policy papers

| Mechanisms of action | Policy papers | | | | | | | |
| --- | --- | --- | --- | --- | --- | --- | --- | --- |
| COM-B | 1 | 2 | 3 | 4 | 5 | 6 | N(%)  Nutr.(%) PA(%) | |
| Psychological capability | 7 | 5 | 5 | 3 | 11 | 3 | 25(28) | 17(18) |
| Physical capability | 1 | 0 | 0 | 0 | 1 | 0 | 1(1) | 2(2) |
| Physical opportunity | 0 | 3 | 7 | 12 | 17 | 8 | 20(24) | 24(27) |
| Social opportunity | 5 | 1 | 4 | 6 | 13 | 8 | 19(22) | 22(24) |
| Reflective motivation | 8 | 1 | 0 | 4 | 9 | 2 | 17(20) | 20(22) |
| Automatic motivation | 0 | 0 | 2 | 5 | 3 | 0 | 4(5) | 6(7) |
| Total | 21 | 10 | 18 | 30 | 54 | 21 | 86(100%) | 91(100%) |
| TDF |  |  |  |  |  |  |  |  |
| Knowledge | 5 | 3 | 5 | 3 | 9 | 2 | 18(17) | 14(13) |
| Skills | 3 | 1 | 3 | 2 | 4 | 1 | 9(9) | 8(7) |
| Social role and identity | 0 | 0 | 0 | 1 | 0 | 1 | 0(0) | 2(2) |
| Beliefs about capabilities | 4 | 0 | 0 | 1 | 1 | 0 | 5(5) | 6(5) |
| Optimism | 0 | 0 | 0 | 0 | 1 | 0 | 1(1) | 1(1) |
| Beliefs about consequences | 4 | 1 | 0 | 3 | 7 | 2 | 12(12) | 13(12) |
| Reinforcement | 0 | 0 | 2 | 4 | 3 | 0 | 4(4) | 5(4) |
| Intentions | 4 | 0 | 0 | 1 | 3 | 0 | 6(6) | 8(7) |
| Goals | 1 | 0 | 0 | 1 | 2 | 0 | 2(2) | 4(4) |
| Memory, attention, and decision processes | 0 | 3 | 1 | 0 | 3 | 0 | 7(7) | 0(0) |
| Environmental context and resources | 0 | 3 | 7 | 13 | 17 | 8 | 17(16) | 24(21) |
| Social influences | 6 | 1 | 4 | 6 | 13 | 8 | 20(19) | 23(21) |
| Emotion | 0 | 0 | 0 | 0 | 2 | 0 | 0(0) | 2(2) |
| Behavioural regulation | 1 | 0 | 0 | 1 | 3 | 1 | 3(3) | 5(4) |
| Total | 28 | 12 | 22 | 36 | 68 | 23 | 104(100%) | 113(100%) |

Policy papers: 1. Principles of good occupational healthcare guide; 2. National nutrition recommendations; 3. Guidelines of the working group to monitor and develop mass catering services; 4. National strategy for physical activity promoting health and well-being 2020; 5. Action plan of the national obesity programme 2012-2015; 6. National recommendations for reduction of sedentary behaviour. Numbers are frequencies. Numbers in parentheses are percentages. Nutr.=nutrition, PA=physical activity. The total is smaller than the sum of Nutr. and PA because parts of the recommendations (e.g. focusing on outcomes) were double coded.
