# Supplementary material for: National policies for the promotion of physical activity and healthy nutrition in the workplace context: a behaviour change wheel guided content analysis of policy papers in Finland
Source: BMC Public Health. 2017 Aug 2;18:87. doi: 10.1186/s12889-017-4574-3 (PMC5540493; doi:10.1186/s12889-017-4574-3)
Supplement: Supplementary file 4 — Identified intervention content recommended in policy papers for nutrition and PA. (DOCX 22 kb) [file 12889_2017_4574_MOESM4_ESM.docx]

Additional file 4

Table S2

Identified intervention content recommended in policy papers for nutrition and PA

| Intervention content | Policy paper | | | | | | | |
| --- | --- | --- | --- | --- | --- | --- | --- | --- |
| Intervention function | 1 | 2 | 3 | 4 | 5 | 6 | N(%)  Nutr.(%) PA(%) | |
| Education | 6 | 3 | 4 | 3 | 8 | 3 | 24(31) | 21(23) |
| Persuasion | 5 | 1 | 1 | 2 | 4 | 2 | 12(16) | 14(15) |
| Incentivization | 0 | 0 | 1 | 4 | 1 | 0 | 3(4) | 4(4) |
| Coercion | 0 | 0 | 0 | 2 | 0 | 0 | 0(0) | 2(2) |
| Training | 1 | 0 | 0 | 0 | 1 | 0 | 1(1) | 2(2) |
| Restriction | 0 | 0 | 0 | 3 | 0 | 2 | 0(0) | 5(5) |
| Environmental restructuring | 0 | 5 | 7 | 11 | 7 | 4 | 20(26) | 25(27) |
| Modelling | 0 | 0 | 0 | 0 | 0 | 1 | 0(0) | 1(1) |
| Enablement | 6 | 1 | 3 | 4 | 9 | 1 | 17(22) | 19(20) |
| Total | 18 | 10 | 16 | 29 | 30 | 13 | 77(100%) | 93(100%) |
| BCT |  |  |  |  |  |  |  |  |
| Goal-setting (behaviour) | 1 | 0 | 0 | 1 | 1 | 0 |  |  |
| Goal-setting (outcome) | 1 | 0 | 0 | 0 | 0 | 0 |  |  |
| Action-planning | 1 | 0 | 0 | 0 | 1 | 0 |  |  |
| Discrepancy between current behaviour and goal | 2 | 0 | 0 | 0 | 1 | 0 |  |  |
| Behavioural contract | 1 | 0 | 0 | 0 | 0 | 0 |  |  |
| Monitoring of behaviour | 1 | 0 | 0 | 1 | 0 | 1 |  |  |
| Feedback on behaviour | 1 | 0 | 0 | 0 | 2 | 0 |  |  |
| Self-monitoring | 0 | 0 | 0 | 0 | 1 | 0 |  |  |
| Self-monitoring of behaviour | 0 | 0 | 0 | 1 | 2 | 1 |  |  |
| Social support (unspecified) | 4 | 1 | 2 | 2 | 8 | 1 |  |  |
| Social support (emotional) | 2 | 0 | 0 | 0 | 2 | 0 |  |  |
| Instructions on how to perform the behaviour | 2 | 5 | 3 | 2 | 6 | 2 |  |  |
| Information about antecedents | 0 | 0 | 0 | 0 | 1 | 0 |  |  |
| Information about health consequences | 1 | 2 | 1 | 1 | 3 | 3 |  |  |
| Demonstration of the behaviour | 0 | 0 | 0 | 0 | 1 | 1 |  |  |
| Prompts/cues | 0 | 1 | 1 | 0 | 1 | 0 |  |  |
| Behavioural practice/rehearsal | 1 | 0 | 0 | 1 | 2 | 3 |  |  |
| Behaviour substitution | 0 | 0 | 0 | 0 | 1 | 1 |  |  |
| Habit formation | 0 | 0 | 0 | 1 | 1 | 3 |  |  |
| Habit reversal | 0 | 0 | 0 | 0 | 0 | 1 |  |  |
| Generalization of target behaviour | 0 | 0 | 0 | 0 | 0 | 1 |  |  |
| Material incentive | 0 | 0 | 0 | 4 | 0 | 0 |  |  |
| Social reward | 1 | 0 | 0 | 0 | 0 | 0 |  |  |
| Reduce negative emotions | 1 | 0 | 0 | 0 | 0 | 2 |  |  |
| Restructuring the physical environment | 0 | 5 | 11 | 9 | 11 | 2 |  |  |
| Restructuring the social environment | 0 | 0 | 0 | 4 | 2 | 5 |  |  |
| Avoidance of exposure to cues for the behaviour | 0 | 0 | 0 | 0 | 1 | 0 |  |  |
| Adding objects to the environment | 0 | 0 | 1 | 2 | 1 | 0 |  |  |
| Identification of self as role model | 0 | 0 | 0 | 1 | 0 | 1 |  |  |
| Incompatible beliefs | 1 | 0 | 0 | 0 | 0 | 0 |  |  |
| Verbal persuasion about capability | 1 | 0 | 0 | 0 | 1 | 0 |  |  |
| Total | 22 | 14 | 19 | 30 | 50 | 28 |  |  |

Policy papers: 1. Principles of good occupational healthcare guide; 2. National nutrition recommendations; 3. Guidelines of the working group to monitor and develop mass catering services; 4. National strategy for physical activity promoting health and well-being 2020; 5. Action plan of the national obesity programme 2012-2015; 6. National recommendations for reduction of sedentary behaviour. Numbers are frequencies. Numbers in parentheses are percentages. Nutr.=nutrition, PA=physical activity. The total is smaller than the sum of Nutr. and PA because parts of the recommendations (e.g. focusing on outcomes) were double coded.
