# Supplementary material for: National policies for the promotion of physical activity and healthy nutrition in the workplace context: a behaviour change wheel guided content analysis of policy papers in Finland
Source: BMC Public Health. 2017 Aug 2;18:87. doi: 10.1186/s12889-017-4574-3 (PMC5540493; doi:10.1186/s12889-017-4574-3)
Supplement: Supplementary file 5 — Frequencies of different targets in recommendations for each policy paper. (DOCX 14 kb) [file 12889_2017_4574_MOESM5_ESM.docx]

Additional file 5

Table S3

Frequencies of different targets in recommendations for each policy paper

| Target | Policy papers | | | | | |
| --- | --- | --- | --- | --- | --- | --- |
|  | 1 | 2 | 3 | 4 | 5 | 6 |
| Individual | 18 | 5 | 5 | 3 | 16 | 10 |
| Community | 0 | 0 | 1 | 3 | 3 | 6 |
| Environment | 0 | 8 | 11 | 10 | 23 | 3 |
| Total | 18 | 13 | 17 | 16 | 42 | 19 |

Policy papers: 1. Principles of good occupational healthcare guide; 2. National nutrition recommendations; 3. Guidelines of the working group to monitor and develop mass catering services; 4. National strategy for physical activity promoting health and well-being 2020; 5. Action plan of the national obesity programme 2012-2015; 6. National recommendations for reduction of sedentary behaviour. Numbers are frequencies.
